# Solitary Rectal Ulcer Syndrome Is Not Always Ulcerated: A Case Report

**DOI:** 10.3390/medicina58081136

**Published:** 2022-08-22

**Authors:** Yi Liu, Zhihao Chen, Lizhou Dou, Zhaoyang Yang, Guiqi Wang

**Affiliations:** 1 Department of Endoscopy, National Cancer Center/National Clinical Research Center for Cancer/Cancer Hospital, Chinese Academy of Medical Sciences and Peking Union Medical College, Beijing 100021, China; dr_jeremy@163.com (Y.L.); m15521121514@163.com (Z.C.); ddx198707@163.com (L.D.); 2 Department of Pathology, National Cancer Center/National Clinical Research Center for Cancer/Cancer Hospital, Chinese Academy of Medical Sciences and Peking Union Medical College, Beijing 100021, China; yzy1060229646@126.com

**Keywords:** solitary rectal ulcer syndrome, endoscopy, magnifying narrow-band imaging (magnifying NBI)

## Abstract

Solitary rectal ulcer syndrome (SRUS) is a benign and chronic disorder well known in young adults that is characterized by a series of symptoms such as rectal bleeding, copious mucus discharge, prolonged excessive straining, perineal and abdominal pain, a feeling of incomplete defecation, constipation and, rarely, rectal prolapse. The etiology of this syndrome remains obscure, and the diagnosis is easily confused with that of other diseases, contributing to difficulties in treatment. We present a case of a 37-year-old male with a nonulcerated rectal lesion grossly resembling a superficial depressed rectal cancer misdiagnosed in another hospital and describe its appearance on endoscopy and in the analysis of its pathological manifestations. The aim of this case report is to report an easily misdiagnosed case of SRUS, which needs to be distinguished from superficial rectal cancer, which should be educational for endoscopists.

## 1. Introduction

Solitary rectal ulcer syndrome (SRUS) is a rare benign rectal disease that is characterized by a combination of symptoms, clinical findings and histological abnormalities [1]. However, SRUS is an infrequent disease that is easily underdiagnosed, with an estimated annual prevalence of one in 100,000 persons. It occurs most commonly in the third decade in men and in the fourth decade in women [2]. Patients mainly exhibit intestinal symptoms, such as constipation, feelings of incomplete defecation, bloody or purulent stools, discomfort with a falling anus and rectal ulcers. Physical examination usually reveals some thickening or a mass typically on the anterior rectal wall. Endoscopy often reveals a discrete, punched-out ulcer. Analysis of the tissue biopsy can confirm the diagnosis. Meanwhile, some medical treatments, including sucralfate, salicylate, corticosteroids, sulfasalazine, mesalazine and topical fibrin sealant, have been reported to be effective [3]. Apart from local medication, the treatment of SRUS also includes the improvement of bowel defecation habits, biofeedback and surgical operation [4]. We report, herein, the case of a 37-year-old male with a nonulcerated rectal lesion grossly resembling a superficial depressed rectal cancer but microscopically proving to be an SRUS. The purpose of publishing this case is to report and discuss the diagnosis of SRUS by magnifying narrow-band imaging endoscopy.

## 2. Case Report

**Figure 1:**
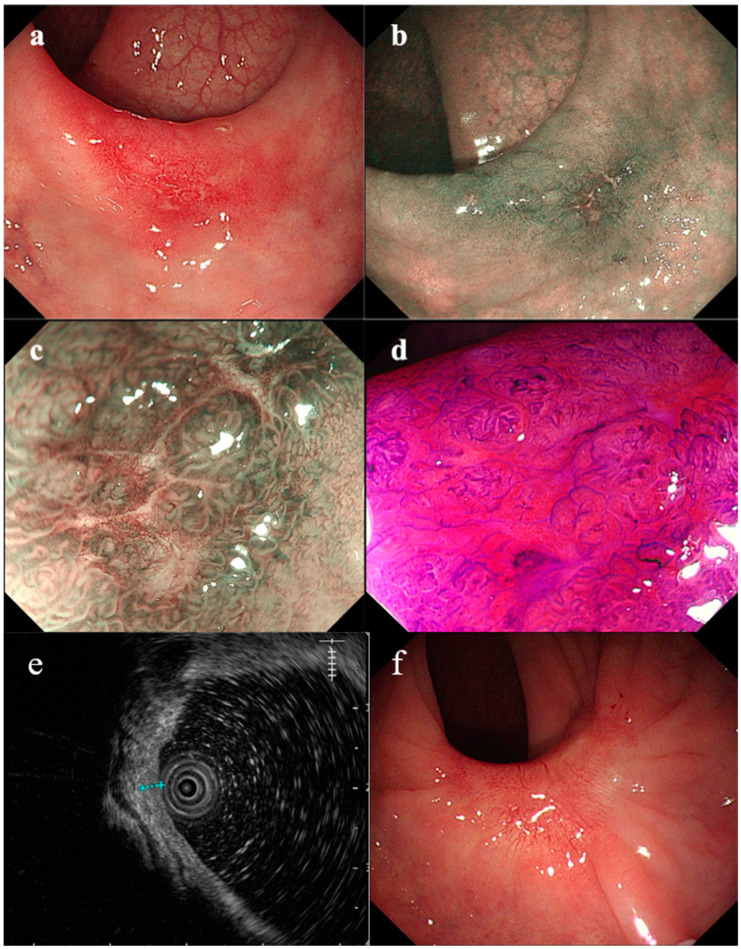
Endoscopic view of rectal lesion: (**a**) white-light endoscopy; (**b**) narrow-band imaging; (**c**) magnified version of the image in (**b**); (**d**) magnified endoscopic view after crystal violet staining; (**e**) the ultrasound image; (**f**) the white-light image of the scar.

**Figure 2:**
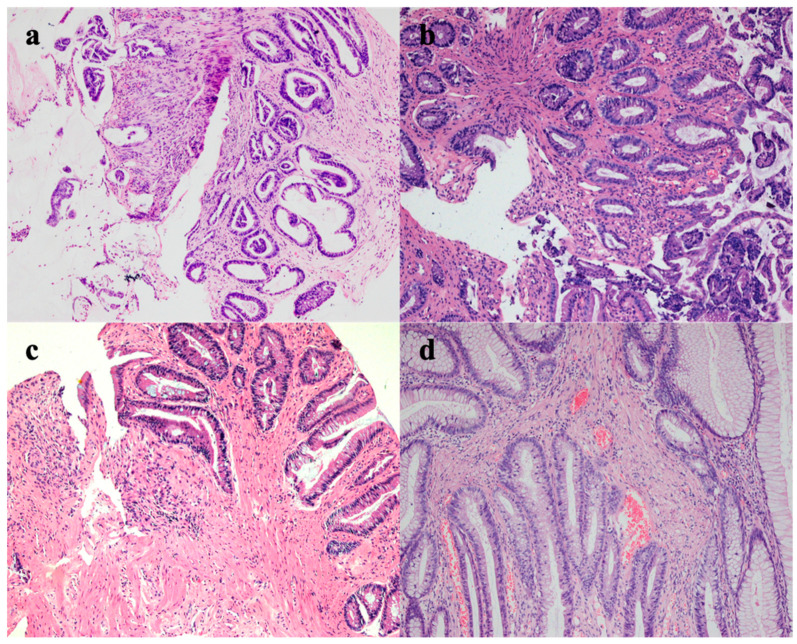
Histologic examination: (**a**–**c**) histologic biopsy in other hospitals: highly distorted and expanded glandular bodies with atypical changes in glandular epithelial cells (magnification: ×100); (**d**) histologic biopsy in our hospital: microvascular hyperplasia and musculomucosal hyperplasia.

**Figure 3:**
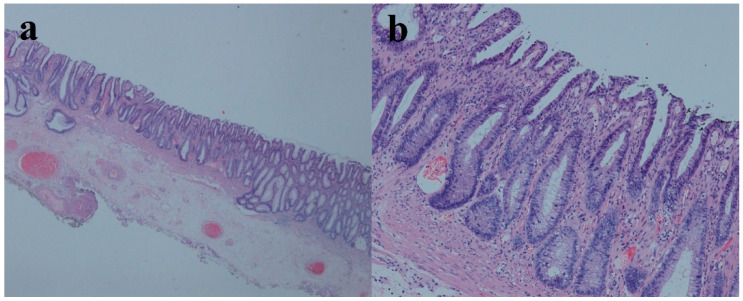
Resection histology: microvascular hyperplasia and musculomucosal hyperplasia. (**a**) Magnification: ×10; (**b**) magnification: ×200.

A 37-year-old male had recurrent abdominal pain, diarrhea and hematochezia for 1 year. The patient had several bad habits such as tobacco use, alcohol consumption and betel quid chewing, and had no previous family history of cancer or special sexual behavior. This patient was recommended to receive endoscopy. Regrettably, he was diagnosed with superficial depressed rectal cancer in other two hospitals. He was then transferred to our hospital in preparation for surgery. The preoperative endoscopy discovered a reddish and irregular but well-defined 0-IIc lesion in the anterior wall of the rectum 4–6 cm from the anal margin (Figure 1a). Magnifying narrow-band imaging revealed fine reticulated vessels with a uniform thickness and distribution (Figure 1b,c). Some irregular pit patterns were observed after crystal violet staining (Figure 1d). Endoscopic ultrasonography (EUS) showed thickening of the mucosal layer at the lesion, and the submucosa was still intact (Figure 1e). Although the morphology of the pit patterns was disordered (Figure 1d), we suspected it not to be an infiltrative tumor, taking the magnifying endoscopic characteristics into account. Thus, we suggested this patient undergo a re-biopsy. Interestingly, histopathological examination at our hospital revealed that the lamina propria was filled with muscle fibers (Figure 2d). However, when pathologists consulted the biopsy results of the external hospital, they found that the glands were highly distorted and enlarged, accompanied by atypical changes in glandular epithelial cells (Figure 2a–c). Therefore, the diagnosis of the patient remained controversial after discussions among experts, with some experts suggesting only inflammatory changes and others suggesting the possibility of high-grade intraepithelial neoplasia. In light of the above situation, we advised the patient to first receive conservative treatment such as a high-fiber diet, reducing irregular stool habits and biofeedback training. The patient still strongly demanded endoscopic submucosal dissection (ESD) for a definitive diagnosis. Finally, the postoperative pathological results supported the diagnosis of SRUS (Figure 3). The wounds recovered well without recurrence, and the symptoms of hematochezia disappeared (Figure 1f).

## 3. Discussion

SRUS is an unusual benign rectal disorder [4,5]. Several etiologies of SRUS have been proposed. This syndrome may have various factors that simultaneously cause the lesions, including rectal prolapse and chronic and severe constipation. Rectal ulcers are frequently described as always being found as single or multiple ulcers located on the anterior wall of the rectum within 10 cm of the anal margin [4]. A relevant study considered that rectal intussusception could lead to localized vascular trauma and, consequently, the onset of solitary local ulceration [6], while other studies showed that uncoordinated muscle contraction in the puborectalis muscle may be associated with increased intra-rectum pressure and pressure in the anal canal, which resulted in ulceration [7,8]. The clinical symptoms include abdominal pain, bleeding, mucus discharge, and chronic and severe constipation, among others. The histological features of SRUS are characterized by a thickening mucosal layer, fibromuscular obliteration, mucous cell proliferation, mucosal gland distortion, etc. [5]. SRUS is easily misdiagnosed as rectal cancer, based on the similarity in the symptomatic profiles and endoscopic features, which include bleeding, mucus discharge, and chronic and severe constipation. To date, these histological features have been helpful in distinguishing SRUS from malignancies. There are few reports about the diagnosis of SRUS by magnifying narrow-band imaging endoscopy.

In this case, the lesion appeared as a nonulcerated rectal lesion, with a superficial depressed area. The patient was misdiagnosed with superficial depressed rectal cancer in other two hospitals. Related studies reported that superficial depressed cancers arose through the de novo pathogenic sequence and had a higher tendency for early invasions [9,10]. As a result of the misdiagnosis, the patient came to our hospital seeking surgery. However, we found that magnifying narrow-band imaging revealed fine reticulated vessels with a uniform thickness and distribution, although some irregular and disordered pit patterns were observed after crystal violet staining. We highly suspected it to be SRUS according to the histopathological examination. We advised the patient to receive conservative treatment, but the patient still strongly demanded endoscopic submucosal dissection (ESD) for a definitive diagnosis. Finally, the postoperative pathological results supported the diagnosis of SRUS.

SRUS is an already well-known but easily misdiagnosed condition; the proper diagnosis and treatment of SRUS remain important challenges. It is worth noting that its rare occurrence usually leads to the fact that it is not properly diagnosed due to the lack of knowledge or lack of experience of doctors. The diagnosis of SRUS can usually be performed by a combination of symptomatology, endoscopy and histology. However, patients sometimes have typical symptoms without typical endoscopic findings. As mentioned above, this lesion did not present with typical ulcerative changes, but presented with superficial depressed changes. We used magnifying NBI and chromoendoscopy to observe this lesion and biopsied again, thus ruling out the possibility of rectal cancer, and finally reached the correct diagnosis. Although there is a little regret due to the fact that the patient strongly demanded ESD for a definitive diagnosis, we believe this is the most fortunate outcome for the patient, as he avoided surgery or even the risk of a permanent fistula.

We consider this case to be a good learning opportunity for gastroenterologists, as when they encounter similar cases, SRUS should be one of the options in the differential diagnosis list.

## 4. Conclusions

Not all SRUS cases present ulcers. Patients with typical symptoms and nonulcerated rectal lesions should be differentiated from those with superficial rectal cancer. Magnifying NBI and chromoendoscopy are useful, and histopathological examination should be performed to confirm the diagnosis.
